# Chief resident behaviors that lead to effective morning reports, a multisite qualitative study

**DOI:** 10.1186/s12909-023-04762-8

**Published:** 2023-10-24

**Authors:** Yihan Yang, Arian Schulze, Amenuve M. Bekui, Sadie Elisseou, Stephanie W. Sun, Seonaid Hay, John P. Moriarty, Stephen R. Holt

**Affiliations:** 1 Washington State University, Internal Medicine Residency Program – Everett, Everett, WA USA; 2 Equity Research and Innovation Center, Yale School of Medicine, New Haven, CT USA; 3 Yale New Haven Hospital, New Haven, CT USA; 4 Harvard Medical School, Boston, MA USA; 5 Massachusetts General Hospital, Boston, MA USA; 6 Yale School of Medicine, New Haven, CT USA; 7 Yale Primary Care Internal Medicine Residency Program, Yale School of Medicine, New Haven, CT USA

**Keywords:** Morning Report, Chief Resident, Graduate Medical Education

## Abstract

**Background:**

Morning report is a fundamental component of internal medicine training and often represents the most significant teaching responsibility of Chief Residents. We sought to define Chief Resident behaviors essential to leading a successful morning report.

**Methods:**

In 2016, we conducted a multi-site qualitative study using key informant interviews of morning report stakeholders. 49 residents, Chief Residents, and faculty from 4 Internal Medicine programs participated. Interviews were analyzed and coded by 3 authors using inductive reasoning and thematic analysis. A preliminary code structure was developed and expanded in an iterative process concurrent with data collection until thematic sufficiency was reached and a final structure was established. This final structure was used to recode all transcripts.

**Results:**

We identified four themes of Chief Resident behaviors that lead to a successful morning report: report preparation, delivery skills, pedagogical approaches, and faculty participation. Preparation domains include thoughtful case selection, learning objective development, content editing, and report organization. Delivery domains include effective presentation skills, appropriate utilization of technology, and time management. Pedagogical approach domains include learner facilitation techniques that encourage clinical reasoning while nurturing a safe learning environment, as well as innovative teaching strategies. Moderating the involvement of faculty was identified as the final key to morning report effectiveness. Specific behavior examples are provided.

**Conclusion:**

Consideration of content preparation, delivery, pedagogical approaches, and moderation of faculty participation are key components to Chief Resident-led morning reports. Results from this study could be used to enhance faculty development for Chief Residents.

**Supplementary Information:**

The online version contains supplementary material available at 10.1186/s12909-023-04762-8.

## Introduction

Morning report is highly valued in internal medicine residency training worldwide [1–5]. In the US, facilitating report represents a substantial portion of Chief Residents’ teaching responsibilities (appendix 1 summarizes the Chief Resident role in graduate medical education) [6, 7]. While many Chief Residents participate in some training to lead report, the amount of report facilitation skills that Chief Residents receive remains unclear [8–11]. With a wide variation in morning report structure across institutions, the literature currently holds no consensus around what constitutes a successful morning report or the skills needed to facilitate report effectively.

Traditionally facilitated by a Chief Resident, morning report is typically a conference room-based didactic venue featuring clinical cases presented under the oversight of attending physicians, often addressing the diagnosis and management of patients whom residents have recently treated [12, 13]. Chief Residents are often responsible for oversight of the case presentation, facilitating discussion around diagnostic and/or management reasoning, and highlighting learning points. Compared to other case-based teaching, report may be a “higher stakes” learning environment and lead to inherent tensions because residents need to explain and potentially defend their recent patient care choices in front of peers and faculty [14, 15]. In exploring learners’ knowledge base, morning report risks embarrassing the resident and may shift emphasis from learning and humanistic care to showmanship and recitation of textbook knowledge [12, 16–18]. Reports’ dynamic, semi-structured format can be challenging to early educators. Morning report must be interactive, allowing both residents and faculty to share their wisdom [2, 19], while also balancing the case presentation and learning objectives with time for open discussion. Chief Residents are also in a unique position during report, leading discussion regarding content in which they are often not expert while also coordinating the input of both junior trainees and senior faculty.

Prior studies have examined residents’ perceptions and expectations of morning report [2, 19, 20], discussed various report formats [2, 13, 15, 17], developed tools for evaluating clinical teaching skills not specific to report [21], or proposed the use of online tools to augment the traditional report format [22]. Others produced conceptual frameworks for facilitating and developing case-based teaching [23–25]. However, previous work has not delineated specific teaching behaviors relevant to the unique morning report setting, especially those led by Chief Residents.

The purpose of this study is to characterize the Chief Resident behaviors that contribute to an effective morning report, as defined by report stakeholders, to optimize the success of morning report in resident education.

## Methods

### Study design and sampling

We conducted a qualitative, constructivist thematic analysis study using key-informant interviews of internal medicine residency program faculty, Chief Residents, and focus groups of residents and interns from March to December 2016. Due to variable styles of morning report, we gathered data at three academic residency programs (Yale Traditional Internal Medicine, Yale Primary Care, Brown Internal Medicine) and one community-based program (Waterbury Internal Medicine), focusing on internal medicine programs due to their high prevalence of morning report relative to other specialties [12].

At the time of this study, report was conducted by Chief Residents in the morning at all sites, lasting 60 minutes, with a mix of unscripted reports (those focusing on currently-admitted patients where the CR is presented a case by a resident and has no prior knowledge of the case details) and scripted reports (cases that are pre-prepared by the chief resident themselves) across the programs. There were no prescribed objectives or morning report “curricula” that Chief Residents were expected to cover during reports. A typical morning report included one Chief Resident facilitator with five to fifteen learners. Yale Traditional and Primary Care programs offered separate morning report for interns and senior residents on two of the weekdays; Brown and Waterbury reports were intended for upper-level residents only. Medical students were invited to attend all reports but were not the target learners at any of the sites. Faculty were present at all sites.

A purposive sample of faculty with expertise in medical education (as recognized by advanced medical education training and/or or recent teaching awards) and current Chief Residents were recruited to participate in interviews. Convenience samples of interns and residents participated in focus groups. The research was approved by the Human Investigation Committee of the Yale University School of Medicine, New Haven, Connecticut. Informed consent was obtained prior to interviews.

### Data collection

An interview guide was developed by the authors and utilized at all sites (appendix 2). The guide was iteratively adjusted as new concepts were identified during data collection. Questions were designed to elicit behaviors that impact the quality of morning report. Interviews and focus groups were conducted using the interview guide until no new concepts emerged.

We conducted six focus groups of four to nine interns and residents per group for a total of 32 residents. Faculty interviews were conducted in-person by five researchers (YY, SS: Chief Residents; AB, SH, SE: faculty) across four training programs. Ten faculty members and seven current Chief Residents were interviewed. To minimize moderator acceptance bias, resident focus groups and Chief Resident interviews were facilitated by a resident research member (YY, SS) at all sites when possible, except Brown University where they were interviewed by a junior faculty member not directly associated with the residency program (SE). All interviews were audio recorded and transcribed.

### Data analysis

Following an inductive, iterative process, we undertook thematic analysis of interviews to identify themes related to Chief Resident behaviors [26–30]. The coding team consisted of three researchers (AB, SH, YY) who reviewed all transcripts independently. Using the constant comparative method, the coding team held regular sessions to reach consensus on transcript codes. The final coding structure was then used to recode all transcripts. Coded texts were compared to identify and analyze concepts until a final thematic structure was developed. Qualitative analysis software NVIVO (Version 11, QSR International, 2015) facilitated data organization and retrieval.

### Reflexivity

Yihan Yang and Stephanie Sun were both rising Chief Residents at Yale Primary Care and Waterbury, respectively, during data collection and analysis and participated actively in their training programs’ morning reports. Remaining authors included associate program directors Stephen Holt (YPC) and Seonaid Hay (Traditional); YPC program director John Moriarty; YPC core faculty Amenuve Bekui, Brown faculty Sadie Elisseou, and a PhD-trained qualitative methods researcher (Arian Schulze).

## Results

Forty-nine participants were interviewed across four internal medicine residency programs (Table 1). Of the ten faculty interviewed, two were program directors.

**Table 1:** Study Participant Demographics, n = 49

**Program**	**n (%)**
Yale Primary Care	14 (28.6%)
Yale Traditional	10 (20.4%)
Waterbury	14 (28.6%)
Brown Traditional	11 (22.4%)
**Participant Level**	**n (%)**
Resident	32 (63.3%)
Chief Resident	7 (14.3%)
Faculty	10 (20.4%)
**Demographics**	**n (%)**
Mean Age (years)	34.2
Female	23 (46.9%)

Four main themes on effective Chief Resident behaviors were identified: (1) Chief Residents should facilitate a comprehensive report that establishes clear learning objectives and anchors case reviews within broader contexts; (2) Chief Residents should adjust modes of delivery and media use to stimulate learner engagement; (3) Chief Residents should employ pedagogical approaches that enhance positive learning environments, promote clinical reasoning, and support innovative, learner-centered strategies; and (4) Chief Residents should moderate faculty involvement in report to prioritize learner participation. Respectively, these themes can be described as relating to: (1) morning report preparation, (2) delivery skills, (3) pedagogical approaches, and (4) faculty participation.

### Chief residents should facilitate a comprehensive report that establishes clear learning objectives and anchors case reviews within broader contexts

All participants identified the importance of preparation prior to report. Learning objectives selection, morning report content curation—including contextualizing cases in their broader fields—and advanced case review were major domains of preparation identified.

Predefined, specific learning objectives enhance the educational value of report: All participants felt that Chief Residents should pre-identify specific learning objectives to serve as the backbone for each report. One resident stated, “When there are very clear, concrete takeaways that we can go use in future practice and seeing patients, that’s really helpful…if there are very clear objectives and very clear aims, it makes [report] a lot better.” Morning reports should be organized to highlight these objectives (Table 2).

**Table 2:** Taxonomy of Chief Resident behaviors that contribute to effective morning reports in internal medicine

**Theme 1: Chief residents should facilitate a comprehensive report that establishes clear learning objectives and anchors case reviews within broader contexts**	
Domain	Behavior example
Predefined, specific learning objectives enhance the educational value of report	• Determine learning points prior to report • Structure morning report to support development of learning objectives
Report content should be curated to reflect a thoughtful selection of cases that are appropriate for target learners and time allotted	• Mix bread and butter and unusual cases • Mix cases focused on differential diagnosis versus management • Do not introduce new ideas in the last five minutes of report
Patient cases should be reviewed in advance	• Has pertinent history, exam findings, lab results, and images ready when asked • Coach residents in advance regarding information to present
**Theme 2: Chief residents should adjust their modes of delivery and media use to stimulate learner engagement**	
Domain	Behavior example
Utilize presentation affect to maximize engagement	• Make eye contact with learners, smile, use humor when appropriate • Embrace silence • Show enthusiasm
Technology and resources are utilized to improve engagement	• Pre-scribe or pre-organize white board for chalk talks • Slides summarize and consolidate content and are not overfilled with text
Time management	• Start and end on time • Allocate adequate time to learning objectives
**Theme 3 – Chief residents should employ pedagogical approaches that enhance positive learning environments, support innovative strategies, and promote clinical reasoning**	
Domain	Behavior Example
Demonstrate effective learner facilitation	• Use warm calling rather than cold calling • Utilize pair-share or small group work • Demonstrate humility, admit “I don’t know” • Invite divergent opinions • Ask learners to describe what they learned
Incorporate innovative teaching strategies	• Use mock debates • Include patients
Promote clinical reasoning	• Ask learners to summarize the clinical syndrome • Introduce “wrinkles” to the case
Provide closure to report cases	• Follow up with residents on results of unsolved cases • Follow up on clinical questions by sending out relevant literature review
**Theme 4: The Chief Resident should moderate faculty involvement in report to prioritize learner participation**	
Domain	Behavior example
Incorporates faculty teaching without losing control of session	• Ask attending perspectives after learners have had a chance to share ideas first • Redirect conversation to learners if attendings are dominating discussion or on a tangent

Report content should be curated to reflect thoughtful case selection that is appropriate for target learners and the time allotted: Participants reported that selection of patient cases for morning report was an important first step in curating appropriate content but had diverging views on the types of cases they preferred. Some favored more clinically relevant, frequently occurring cases:

*“Tell me about the bread and butter. I want my intern to know what the heck he’s going to do about a diabetic foot ulcer…I don’t want to know about the other weird rare stuff that none of the attendings who exist in the room have seen.” - Resident*.

Others preferred cases with uncommon presentations. Senior residents voiced preference for cases that emphasized medical decision making and management. Several participants noted reports which highlight nontraditional topics like medical ethics, community engagement, and care discussion goals as particularly impactful:

*“Reports that stick out in my mind are the ones that have a bigger picture to them, even beyond the case that was presented. Whether it’s medicine as a field or something ethical, I think those are the most engaging.” - Resident*.

Faculty preferred intake reports on patients admitted the night prior; Chief Residents and residents did not. Participants suggested variation of style, content, and delivery (see Table 2) throughout the year.

Many participants cautioned against overloading morning report content. As teaching points accrue, learners face an increasing cognitive load, taking less away from morning report. One faculty participant suggested, “One should stop delivering new concepts within five minutes of the report’s end.”

Patient cases should be reviewed in advance: A Chief Resident’s familiarity with the case leads to a more effective report (Table 2):

*“I think preparation on the part of the chief is super important…They don’t know these patients. They didn’t admit them, but they do a ton of chart digging and really made sure they knew their patients well and thought about their teaching points.” -Resident*.

If residents present the case, participants stated that Chiefs should coach residents to streamline the presentation and teaching points.

### Chief residents should adjust modes of delivery and media use to stimulate learner engagement

Participants spent much of their interviews describing content delivery behaviors that aid in a successful morning report. Domains of effective delivery include: (1) engaging presentation affect, (2) technology and resource utilization, and (3) time management.

Utilize engaging presentation affect to maximize engagement: Affect skills include the appropriate use of verbal and non-verbal cues such as making eye contact, modulating speech and tone, showing enthusiasm through body language, using humor when appropriate, and embracing occasional silence. One resident reported, “It’s definitely energy and positivity that I think really sets the tone for the whole report. If you have…energy and excitement, you’re like, ‘Guys, this is a great case.’” Taking advantage of the physical space can enhance engagement, such as reorganizing the seating or walking around the room to narrow the gap between teacher and learners.

Technology and resources should be utilized to improve engagement: Chief Residents could consider ways of using multimedia that enhances, not detracts from, teaching (Table 2). Slides should be used sparingly to consolidate teaching points; text-heavy presentations were ineffective. For chalk talks, organizing the white board before morning report begins helps orient learners to predetermined learning objectives. A resident added,

“I think the board work is also an advantage, a positive, because it keeps it interactive. It keeps the audience at the same pace as the presenter, whereas if you’re at a lecture, if you get a page, if you lose the ability to follow along in that moment, sometimes it can be hard to get back.”

Adequate time should be allocated to the learning objectives: Many participants stressed the importance of time management, including starting and ending morning report on time to demonstrate respect for participants. One faculty member emphasized: “Not being able to manage the clock actually is a big [flaw]. Spending too much time on the history and then not being able to focus on another part of the case.” Chief Residents should also spend an appropriate amount of time on each learning objective.

### Chief residents should employ pedagogical approaches that enhance positive learning environments, support innovative strategies, and promote clinical reasoning

Participants underscored the importance of learner-centered interactions and focused on the following four domains: (1) effective learner facilitation skills, (2) incorporation of innovative teaching techniques, (3) promotion of clinical reasoning, and (4) provision of case closure.

Demonstrate effective learner facilitation skills: As reports are meant to be interactive, Chief Residents should serve as facilitators rather than lecturers. Establishing a safe report learning environment is essential. Specific behaviors included using learners’ names, creating smaller groups to encourage participation, and warm calling (notifying a learner in advance that they will be asked a question) (Table 2):

*“When we are grouped into three, for example…and then you give your opinion, it’s a lot easier…It’s our group coming up with a certain notion. Which may get shot down, but it feels a lot better because it’s a group effort and not me. I think that is one way to balance out that awkwardness of being around attendings and being around people who do affect your progression through your residency.” - Resident*.

Participants emphasized facilitating report in a learner-centered fashion without losing control of the session: engaging all learners, encouraging peer teaching, and actively “diagnosing” learners utilizing appropriate and varied question types. While participants recommended predetermined learning objectives, they also advised Chief Residents to remain flexible, adjusting objectives and teaching strategies if learners are not responding as anticipated:

*“If [they’re] coming up with a differential and no one’s raising their hand or speaking up…and maybe they’ll start it or prompt ideas for people to think of, which line of thought we should be going down…They should be able to recognize those pauses and help out.” - Faculty*.

Chief Residents should allow discussion of learner-identified objectives that may be unanticipated if the topic is enriching for the group but provide gentle redirection to avoid uninformative tangents.

Learning consolidation was also identified as key to a successful report. Chief Residents share many teaching pearls during report; reviewing or applying knowledge at the end can help with retention:

*“When I was an intern, [chiefs] would have a [board review] question at the start of the morning report that was about the topic from the day prior so that was sort of like giving people a quick reminder about the thing we talked about yesterday.” – Chief Resident*.

Residents appreciated those who provided handouts, literature, or advance organizers that could be referenced during clinical care.

Incorporate innovative teaching strategies: As morning report generally follows the same format within programs, Chief Residents who are innovative were considered particularly successful. Examples include a brief physical exam skills workshop, role play, mock debates, or inviting the patient to report. Participants also identified using games—trivia quizzes or a diagnostic competition between interns, residents, and attendings—as an effective teaching strategy:

*“Right now what we do is the last morning report of the month before the residents rotate off we do a jeopardy and all of the questions are about the topics that we did in the previous month. Each category will be basically one of the cases that we did…People seem to like that and it’s a good end of the month, relaxed fun thing.” – Chief Resident*.

Promote clinical reasoning: Promotion of clinical reasoning is critical to a successful report. Several participants highlighted asking learners to provide a summary of the patient’s clinical syndrome, “the one-liner,” challenging learners to identify the pertinent details of the patient’s presentation. Additional behaviors identified include mixing low-level recall questions with higher-order analytic questions, role modeling clinical decision making, and introducing step-wise nuances of the case that alter diagnostic or management considerations:

*“He started introducing wrinkles. What about if this was there? How do you think about this? How does your differential change? Even though we know the patient on the board was pretty otherwise wrinkle-free it forced us to think about a relatively common admission in a different way.” -Resident*.

Chief Residents should provide closure to report cases: report often ends with clinical questions that remain unanswered or with the patient case unsolved. Several residents described feeling unsatisfied when reports—particularly intake—lacked closure. One resident recalled, “Coming back the next day and saying, ‘Yesterday, we talked about patient x. Here’s a follow-up’… I think it adds just that extra element that makes being [at report] that day important.” Chief Residents who follow-up on clinical questions by distributing relevant literature or by updating residents on the patient’s outcome later in the week were seen as role models for self-directed learning.

### Chief residents should moderate faculty involvement to prioritize learner participation

The role of faculty at morning report was a controversial topic among all participants. Residents, Chief Residents, and faculty alike acknowledged that the presence of faculty often affects morning report dynamics:

*“When [faculty are] demonstrating non learner-centered behaviors, then I think that’s when they need to be corralled in some way. But if they’re actually adding to the case, then I think they should be allowed to speak. Maybe that’s why it’s so hard…because it has to be judged in the moment.” -Faculty*.

To balance the benefit of faculty perspectives, participants suggest that Chief Residents open discussion to attendings only after learners have had a chance to share their ideas. Chief Residents should redirect conversation back to learners if attendings are dominating the discussion:

*“I think it’s a tough role for them because they’re in their Chief Resident role between the residents and the faculty. They’re trying to respect the faculty. When the faculty starts talking, you can’t really cut them off. You can see the chiefs really do try to get it back to the residents, I think, most of the time.” -Resident*.

Some Chief Residents discuss expectations with attendings prior to report and provide them with specific tasks that would be helpful to the discussion. However, some pointed out the value of faculty carefully interjecting their expertise when appropriate:

*“If the group is floundering a little bit or missing a key point, [attendings] can interject bits of information, like clinical pearls or clinical reasoning, things that are being missed, but not take over and lecture.” -Resident*.

## Discussion

Using semi-structured interviews of residents, Chief Residents, and faculty from four internal medicine residency programs, we identified four themes of Chief Resident behavior that produce successful morning reports: preparing content with clear learning objectives, using presentation skills and media resources that are conducive to engagement, incorporating pedagogical approaches that promote clinical reasoning in a positive learning climate, and moderating faculty participation.

While some of the identified behaviors (e.g. adequate preparation and defining clear learning objectives) are well-suited to teaching in any setting, many of the behaviors identified highlight the differences between report and other clinical education settings. In contrast to the passive learning that might occur at a traditional noon conference or grand rounds, a successful report necessitates strategies that encourage engagement of multiple learner levels and promotion of clinical reasoning.

Several of our themes’ domains are consistent with research that emphasizes the importance of safe learning climate, small group learning, teacher humility, and review of previously presented material to promote memory consolidation in education [31–34]. To our knowledge, this study is the first to demonstrate how these pedagogical principles translate to morning report teaching behaviors, particularly reports led by Chief Residents.

A recent multi-site study on morning report suggests a movement towards predominantly prepared rather than “intake” or “unscripted” reports, a focus on “rare” and/or “severe” cases, a lack of attendance by specialist faculty, as well as a dominant use of slide presentations and reliance on “open-ended questions” without much use of small groups for learner engagement [13]. Our study suggests that prepared reports were preferred by learners while faculty felt that intake reports were more effective. This may be an area for additional research. Programs should also consider their case selection for report, as our participants felt that Chief Residents should choose a mix of cases ranging from bread and butter, to “zebra,” to non-traditional topics like medical ethics. Based upon results from our study, we also encourage Chief Residents to consider inviting specialists to report and utilizing engagement strategies outside of slide presentations and traditional “open-ended” questions.

Our study has limitations. Morning report is not unique to internal medicine programs; the results from this study may not be generalizable to all specialties. The residency programs studied are located in New England. However, the characteristics of morning reports at the four programs in this study are similar to those recently described in the multi-site study by Heppe et al. in terms of duration, number of cases, primary facilitator, and attendees [13]. Additionally, all three members of the coding team were part of a single residency program (YPC) and may have been influenced by their program’s expectations regarding morning report. The coding team did strictly adhere to an inductive, iterative process to limit the influence of any pre-identified hypotheses, and the findings were reviewed and approved by research members across all sites. We also chose to exclude medical students in our interviews as they were not the primary target learners for morning report in any of the participating programs, although their attendance was encouraged. We acknowledge that medical students attend morning report with a different fund of knowledge and need for psychological safety compared to residents, and that student opinions might have shed light on some aspects of report. Finally, our study was conducted prior to the COVID-19 pandemic and the movement of didactics to online learning. However, we believe the principles for our four themes are transferrable to morning report conducted in the virtual space, with utilization of technology to enhance learner engagement becoming even more important (for example, use of polling, chat, annotation, break-out rooms, and white board for chalk talks).

Based on the sheer number of behaviors identified, facilitating a successful morning report is clearly a complex task for even the most experienced educators. Programs committed to maximizing the potential of morning report for resident education should invest in training their rising Chief Residents. Learning to teach effectively necessitates deliberate practice, mentorship, and feedback. Future research should incorporate the themes described above as a framework to both train and evaluate Chief Resident competency in leading morning report.

## Conclusion

This study is the first to fully characterize the specific Chief Resident skills required to conduct morning report successfully. Residency programs should train their Chief Residents to incorporate those behaviors associated with successful morning reports.

### Electronic supplementary material

Below is the link to the electronic supplementary material.

Supplementary Material 1

## Data Availability

The datasets used and/or analyzed during the current study are available from the corresponding author upon request.
