# Supplementary material for: Chief resident behaviors that lead to effective morning reports, a multisite qualitative study
Source: BMC Med Educ. 2023 Oct 24;23:789. doi: 10.1186/s12909-023-04762-8 (PMC10598973; doi:10.1186/s12909-023-04762-8)
Supplement: Supplementary file 1 — Supplementary Material 1 [file 12909_2023_4762_MOESM1_ESM.docx]

**Appendix 1**

**Description of the Chief Resident Role in Graduate Medical Education**

In many countries, chief residency is a role that is well established in the culture of graduate medical education. Depending on the program and specialty, one or more chief residents per program each year can be selected to serve out their “chief year” during either the last year of specialty graduate medical training, or to serve an extra year after their full specialty training is completed(36). Chief residents are often selected by faculty or colleagues based upon their reputation for clinical or educational prowess. The selection process and criteria are often opaque and variable by program(37). There are no specific training or licensure requirements in order to serve in the chief resident role. The roles and responsibilities of chief residents are defined by each training program’s leadership and are therefore quite heterogenous. However, the chief year usually consists of some combination of clinical service, didactic and/or clinical teaching, and graduate medical education program administration tasks(38, 39).

**Appendix 2**

**Open-Ended Interview Template***

- What are the goals of morning report?
  - Why do you feel these goals are important for this particular setting?
  - How do goals of morning report differ from other teaching settings?
- What are the characteristics of an outstanding morning report?
- What challenges serve as barriers to an outstanding morning report?
- What Chief Resident behaviors lead to a successful morning report?
  - what are some specific examples of these behaviors that you have seen, and why have they been successful?
- What Chief Resident behaviors lead to an unsuccessful morning report?
  - What are some specific examples of these behaviors that you have seen, and why have they been unsuccessful?
- What do you think is the role for attendings during morning report?

*Detailed answers to the first and last question are further explored in additional manuscripts.
